# The Value of Blood Oxygenation Level-Dependent (BOLD) MR Imaging in Differentiation of Renal Solid Mass and Grading of Renal Cell Carcinoma (RCC): Analysis Based on the Largest Cross-Sectional Area versus the Entire Whole Tumour

**DOI:** 10.1371/journal.pone.0123431

**Published:** 2015-04-15

**Authors:** Guang-yu Wu, Shi-teng Suo, Qing Lu, Jin Zhang, Wan-qiu Zhu, Jian-rong Xu

**Affiliations:** 1 Department of Radiology, Renji Hospital, Shanghai Jiao Tong University School of Medicine, Pudong, Shanghai, China; 2 Department of Urology, Renji Hospital, Shanghai Jiao Tong University School of Medicine, Pudong, Shanghai, China; Affiliated Hospital of North Sichuan Medical College, CHINA

## Abstract

**Objectives:**

To study the value of assessing renal masses using different methods in parameter approaches and to determine whether BOLD MRI is helpful in differentiating RCC from benign renal masses, differentiating clear-cell RCC from renal masses other than clear-cell RCC and determining the tumour grade.

**Methods:**

Ninety-five patients with 139 renal masses (93 malignant and 46 benign) who underwent abdominal BOLD MRI were enrolled. R2* values were derived from the largest cross-section (R2*_largest_) and from the whole tumour (R2*_whole_). Intra-observer and inter-observer agreements were analysed based on two measurements by the same observer and the first measurement from each observer, respectively, and these agreements are reported with intra-class correlation coefficients and 95% confidence intervals. The diagnostic value of the R2* value in the evaluation was assessed with receiver-operating characteristic analysis.

**Results:**

The intra-observer agreement was very good for R2*_largest_ and R2*_whole_ (all > 0.8). The inter-observer agreement of R2*_whole_ (0.75, 95% confidence interval: 0.69~0.79) was good and was significantly improved compared with the R2*_largest_ (0.61, 95% confidence interval: 0.52~0.68), as there was no overlap in the 95% confidence interval of the intra-class correlation coefficients. The diagnostic value in differentiating renal cell carcinoma from benign lesions with R2*_whole_ (AUC=0.79/0.78[observer1/observer2]) and R2*_largest_ (AUC=0.75[observer1]) was good and significantly higher (p=0.01 for R2*_largest_[observer2] vs R2*_whole_[observer2], p<0.01 for R2*_whole_[observer1] vs R2*_largest_[observer2]) than R2*_largest_ for observer 2 (AUC=0.64). For the grading of clear-cell RCC, both R2*_whole_ and R2*_largest_ were good (all > 0.7) and were not significantly different (p=0.89/0.93 for R2*largest vs R2*whole[observer1/observer2], 0.96 for R2*whole[observer1] vs R2*largest[observer2] and 0.96 for R2*whole [observer2] vs R2*largest[observer1]).

**Conclusions:**

BOLD MRI could provide a feasible parameter for differentiating renal cell carcinoma from benign renal masses and for predicting clear-cell renal cell carcinoma grading. Compared with the largest cross-section, assessing the whole tumour provides better inter-observer agreement in parameter measurement for differentiating renal cell carcinoma from benign renal masses.

## Introduction

Renal cell carcinoma (RCC) accounts for 3% of all adult malignancies and is the most lethal urogenital tumour [1]. The majority of renal masses require evaluation through imaging modalities, and accurate discrimination focuses on separating surgical renal masses from nonsurgical renal masses to avoid unnecessary iatrogenic trauma [2]. In addition, the pre-operative identification of RCC subtypes is an important goal for imaging evaluation because different RCC subtypes display unique histopathological features, gene expression patterns, and clinical behaviours. The results of previous studies have suggested that patients with chromophobic or papillary RCC have a better prognosis than patients with clear-cell renal cell carcinoma (ccRCC) [3]. Moreover, using imaging modalities to determine the tumour grade is also useful in the clinic because it is increasingly difficult to obtain accurate histological diagnoses with the recent advances in percutaneous minimally invasive techniques, radiofrequency ablation (RA) and active surveillance protocols [4,5].

Contrast-enhanced CT and MRI have recently become two of the most commonly used modalities for assessing renal lesions, allowing for the accurate diagnosis of RCC in most cases. However, CT and MRI features cannot reliably distinguish oncocytoma and fat-free angiomyolipoma from malignant renal neoplasms [6]. Moreover, contrast-induced nephropathy due to contrast-enhanced CT [7] and the conflict of the temporal resolution, spatial resolution and scanned slices exhibit limited accuracy in the quantification of the haemodynamics of contrast-enhanced MRI for the evaluation of renal masses. Alternatively, blood oxygenation level-dependent (BOLD) MRI has been used as a rapid, non-invasive method for assessing regional tissue oxygen concentrations using the paramagnetic properties of deoxyhaemoglobin as an endogenous contrast agent because the increased deoxyhaemoglobin concentration in the blood will lead to a decreased T2* relaxation time of protons [8, 9], based on which the rate of spin dephasing (R2*; equal to 1 / T2* relaxation time) can be calculated and used in the assessment of renal masses [9]. However, a major concern is that the diagnostic value of BOLD MRI in renal mass evaluation has not been determined, which is important for its clinical application. Furthermore, the difference between various assessment methods based on the largest cross-section and the whole tumour regarding R2* values of the renal mass has not yet been discussed. The objective of our study was to study the value of assessing renal masses using different methods in parameter approaches and to determine whether BOLD MRI is helpful in differentiating RCC from benign renal masses, differentiating ccRCC from renal masses other than ccRCC and determining the tumour grade.

## Materials and Methods

This is a single-institution study approved by the Shanghai Jiao Tong University School of Medicine Institutional Review Board and was performed in accordance with the ethical guidelines of the Declaration of Helsinki. Written informed consent was obtained for each patient. Patients were enrolled with the following eligibility criteria: 1) patients underwent abdominal MRI, including BOLD MRI, between January 2010 and February 2012; 2) at least one renal mass was observed on the MRI of the patients. In cases with cystic components within the renal mass, cases were enrolled only if the diameter of the solid component was >1 cm (because of the limited spatial resolution of BOLD MRI scans); and 3) renal masses were pathologically confirmed at our institution, and masses suspected to be benign were followed for at least 18 months.

### MR imaging

Patients were examined with a 3.0-T MR scanner (Signa HDxt, GE Healthcare, Milwaukee, WI, USA) with an eight-channel torso phased array coil. Patients were imaged in the supine position with the following sequences: 1) transverse breath-hold in and opposed phase spoiled gradient echo (3.9 ms/2.4,1.2 ms/4 mm/1 mm/40 cm [repetition time/echo time/section thickness/intersection gap/field of view]); 2) transverse respiratory-triggered T2-weighted fast spin echo with fat suppression (7000 ms/100 ms/5 mm/0 mm/40 cm); 3) coronal breath-hold single-shot fast spin echo (∞/100 ms/5 mm/0 mm/44 cm); 4) transverse multi-gradient-recalled-echo (16 echos) sequence with breath-hold (70 ms/2.3–51.4 ms/5 mm/0 mm/40 cm); and 5) transverse breath-hold three-dimensional T1-weighed spoiled GRE with fat suppression (3.3–3.7 ms/1.4–1.6 ms/3-4 mm/0 mm/40-45 cm) before intravenous injection and with multi-phase imaging after intravenous injection (at the corticomedullary, nephrographic, and excretory phases, respectively) of Gd-DTPA (0.1 mmol/kg of body weight; Magnevist, Bayer Schering Pharma).

### Image Evaluation

The imaging data provided during the image evaluation were collected and organised by observer 3, who had 4 years of experience in abdominal MR imaging. All of the imaging data were evaluated using a home-developed software based on Matlab (Mathworks, Natick, Mass). Background noise was subtracted from the liver signal intensity, and the net value was plotted against the echo time for each image. The signal intensity drop was fitted on a pixel-by-pixel basis to a monoexponential decay using a least-squares fit method, which was described in a previous study [10]. In addition, T2* was transformed into reciprocal R2*: R2*[Hz] = 1000/T2*[ms].

All images were independently analysed by observers 1 and 2 (each with more than 5 years of experience in abdominal radiology), who were informed of the number and location of renal masses but were blinded to the patients' clinical history and outcomes.

**Fig 1:**
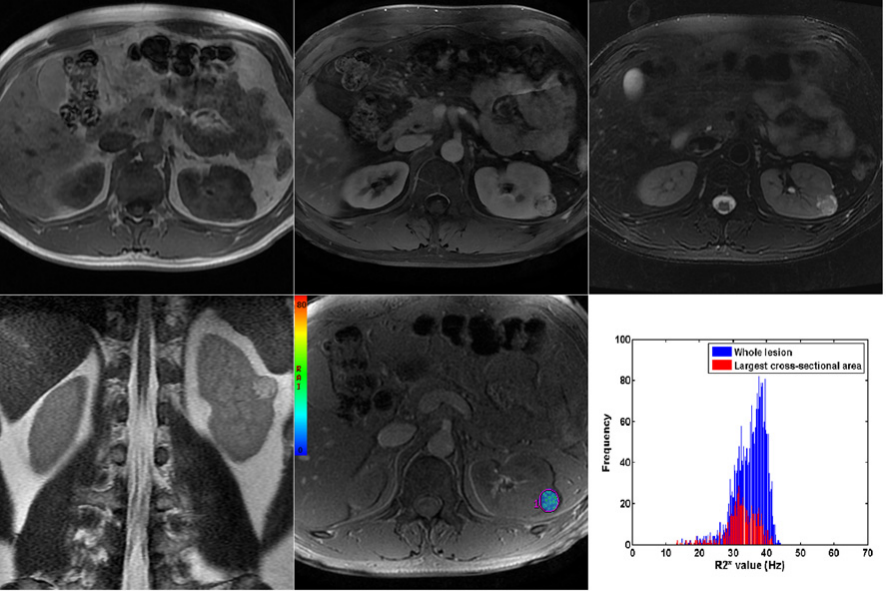
MRI scans of cRCC. (A) unenhanced T1-weighted in-phase (B) contrast-enhanced T1-weighted with fat suppression at nephrographic phase (C) T2-weighted image with fat suppression (D) coronal T2-weighted image (E) colour rate of spin dephasing (R2*) map, mean R2* value of renal cell carcinoma and histogram of R2* value derived based on largest cross-section and the whole tumour (F).

**Fig 2:**
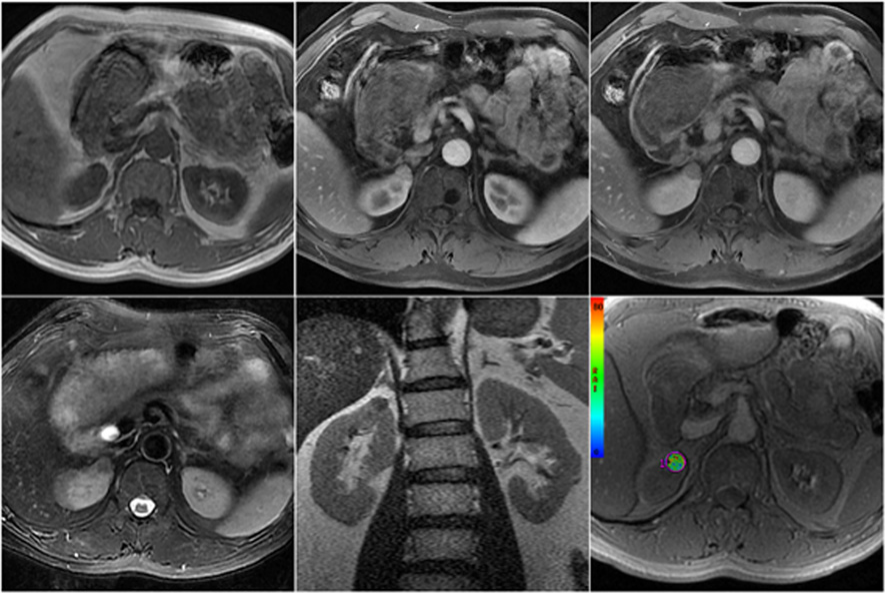
MRI scans of angiomyolipoma. (A) unenhanced T1-weighted in-phase (B) contrast-enhanced T1-weighted with fat suppression at corticomedullary phase (C) contrast-enhanced T1-weighted with fat suppression at nephrographic phase (D) coronal T2-weighted image (E) T2-weighted image with fat suppression (F) colour rate of spin dephasing (R2*) map, mean R2* value

Quantitative analysis was performed by placing regions of interest (ROIs) on the images of R2*. The ROIs were manually drawn to encompass as much of the renal mass as possible to highlight the entire enhancing portion of the solid component on the largest cross-section, excluding the necrotic portion and avoiding adjacent structures. All of the slices of the renal mass were obtained. The ROIs included at least 15 pixels (mean, 85.7 pixels; range, 15 ~ 227 pixels) to consider the ROI as reliable. Routine contrast-enhanced MRI scans were used as anatomical references for the ROIs, and the discontinuous area was also measured. The ROIs were chosen to be representative of the tissue being evaluated (Figs 1 and 2). On in-phase and out-of-phase T1-weighted imaging, a focal fatty component (not diffuse fatty tissue) identified in the solid lesion was also included in the measurements.

The analysis proceeded with patients being recruited between January 2010 and February 2012. All of the data were evaluated twice with a one-month interval by both observers, and the R2* value based on the largest cross-section (R2*_largest_) and the whole tumour (R2*_whole_) from the renal mass were collected. Intra-observer agreement was analysed based on the two measurements by the same observer, and inter-observer agreement was based on the first measurement from each observer. In addition, the values measured with the first measurement from each observer were used for the statistical model to classify subjects with the cut-off values calculated by receiver-operating characteristic (ROC) analysis and were used to explore the diagnostic value of BOLD imaging in evaluating renal masses.

### Reference Standard

All RCCs were confirmed by histopathological examination after total or partial nephrectomy, and lesions that were stable for at least 18 months on follow-up MR images (obtained either before or after the MR images were evaluated) with no suspicious findings on contrast-enhanced images were presumed to be benign. The grading of ccRCC was assessed with the Fuhrman system, which stratifies the tumour grade based on the size and shape of the nuclei and on the prominence of the nucleoli; all ccRCCs were categorised into four grades (Grades I–IV). On the basis of the Fuhrman nuclear grade, all cases were merged into low- (Grade I+II) or high-grade groups (Grade III+IV).

### Statistical Evaluation

Statistical analysis was based on each renal mass. Statistics were calculated using Medcalc (Version 12.0.4). Differences in the R2* values of different groups were analysed by the Mann-Whitney U test. Inter-observer and intra-observer agreements were assessed using the proposed method using intra-class correlation coefficients (ICCs) [11], and Bland-Altman plots [12] were also constructed. ICC values of 0–0.20, 0.21–0.40, 0.41–0.60, 0.61–0.80, and 0.81–1.00 were considered to indicate poor, fair, moderate, good, and very good agreement, respectively. ROC analysis was used to determine the cut-off value derived from the subjects, and the area under curve (AUC) was calculated to compare the diagnostic value in differentiating RCC from benign masses, ccRCC from other RCC types and different ccRCC tumour grades. The diagnostic value of the parameter was defined as “poor” when the AUC was between 0.5–0.7, “good” when the AUC was between 0.7–0.9 and “excellent” when the AUC was larger than 0.9. A p value less than 0.05 was considered statistically significant.

## Results

Three patients were excluded because of severe artefacts, poor differentiation between the kidney and adjacent structures, poor visualisation of the cortex and medulla or images that were inappropriate for calculating R2* value. Finally, the remaining 95 patients (gender, 56 male and 39 female; mean age, 67.1 years; range, 26–82 years) with 139 renal masses (mean diameter, 22.5 ± 14.6 mm; range, 11.7–82.4 mm) formed the study population. Of the 139 renal lesions, 91 were malignant (69 ccRCCs, 17 papillary RCC, 4 chromophobe RCC and one urothelial cancer), and 16 were benign renal lesions (14 AMLs, one renal fibroma and one oncocytoma), as confirmed by histopathology. The rest of the patients (29 patients with 32 masses) were followed-up with conventional MRI. Of these, 2 patients with 2 renal masses appeared to have an increase in tumour size and were confirmed to have ccRCC in a subsequent surgery.

**Fig 3:**
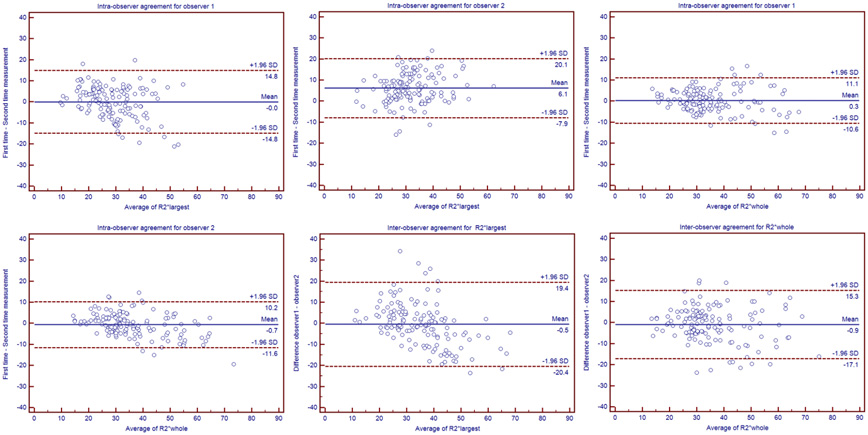
Bland-Altman plots for intra- and inter-observer agreement of R2* measurements based on the largest cross-section and the whole tumour with mean absolute differences (continuous line) and 95% CI of the mean differences (dashed lines). For intra-observer agreement, measurements from both the first and second MRI scan were included in this analysis. While for the inter-observer agreement, the Bland-Altman plot shows the difference between measurements of two observers against the average measurement. (A) Intra-observer agreement of R2*largest for observer 1. (B) Intra-observer agreement of R2*largest for observer 2. (C) Intra-observer agreement of R2*whole for observer 1. (D) Intra-observer agreement of R2*whole for observer 2. (E) Inter-observer agreement for R2*largest. (F) Inter-observer agreement for R2*whole.

The R2*_largest_ and R2*_whole_ values of the renal masses measured by observer 1 and observer 2 are listed in Table 1. Both R2*_largest_ and R2*_whole_ were significantly higher in benign lesions than in RCC, whereas the difference between ccRCC and renal masses other than ccRCC were not significant. For the grading of ccRCC, both R2*_largest_ and R2*_whole_ were significantly higher in high-grade ccRCC than in low-grade ccRCC. The differences between the intra- and inter-observer measurements are listed in Table 2, and the corresponding Bland-Altman plots are shown in Fig 3. For the inter-observer agreement, the R2*_whole_ value resulted in good agreement, with an ICC of 0.75, and the ICC was significantly improved when using R2*_whole_ values instead of R2*_largest_ values, as there was no overlap in the 95% confidence interval. The difference in the observed ICC between the R2*_largest_ and R2*_whole_ values was statistically significant (p<0.05). The intra-observer agreement of R2*_largest_ and R2*_whole_ were both classified as ‘very good.’

**Table 1:** The R2*_largest_ and R2*_whole_ values calculated by both observers.

	R2*_largest_		R2*_whole_	
	Observer 1	Observer 2	Observer 1	Observer2
**RCC**	31.97±8.16	27.14±11.16	32.30±7.44	30.08±7.59
**Benign lesion**	40.14±11.16	34.17±13.33	43.51±13.78	40.07±12.23
**p value** ^1^	< 0.01	< 0.01	< 0.01	< 0.01
**ccRCC**	33.77±7.68	29.70±6.13	35.16±7.55	31.27±7.57
**Renal mass other than ccRCC**	35.55±12.73	28.86±10.65	37.30±14.20	35.98±13.61
**p value** ^2^	= 0.16	= 0.86	= 0.19	= 0.08
**High-grade ccRCC**	38.70±9.08	33.12±7.18	36.75±8.22	38.16±9.78
**Low-grade ccRCC**	32.20±5.99	28.36±5.20	29.68±4.07	32.20±5.86
**p value** ^3^	< 0.01	< 0.01	< 0.01	< 0.01

^1^. p value for difference of R2* value between groups of RCCs and benign lesions.

^2^. p value for difference of R2* value between groups of ccRCCs and renal masses other than cRCC.

^3^. p value for difference of R2* value between groups of high-grade and low-grade cRCCs.

**Table 2:** Consistency of the parameter measure procedure.

	Intra-class correlation coefficient	95% confidence-interval	
		Lower Bound	Upper Bound
**Intra-observer**			
**Observer 1**			
R2*_largest_	0.82	0.78	0.83
R2*_whole_	0.86	0.80	0.89
**Observer 2**			
R2*_largest_	0.81	0.73	0.86
R2*_whole_	0.81	0.79	0.84
**Intra-observer**			
R2*_largest_	0.61	0.52	0.68
R2*_whole_	0.75	0.69	0.79

**Fig 4:**
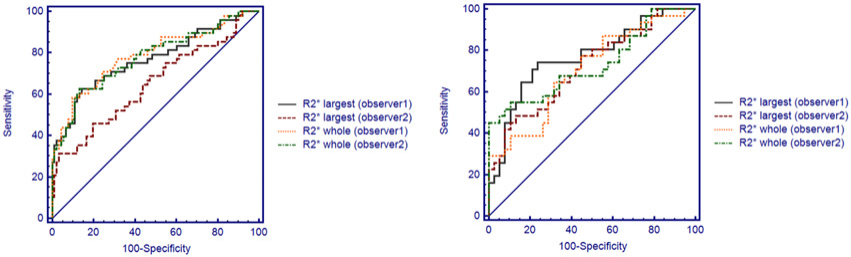
The ROC curve for R2*largest vs R2*whole for renal masses evaluation. (A) Differentiating RCC from benign renal masses. (B) Differentiating high grade RCC from low grade.

The performance of the R2* value in differentiating RCC from benign lesions and ccRCC from renal masses other than ccRCC is listed in Table 3, and the ROC curves are shown in Fig 4. Discrimination of the clear-cell renal cell carcinoma grade was also possible. Regarding the differentiation of RCC from benign lesions, the diagnostic values of R2*_whole_ for both observers (AUC = 0.79/0.78[obserber1/observer2]) and R2*_largest_ for observer 1 (AUC = 0.75) were good, while R2*_largest_ for observer 2 was poor (AUC = 0.64). Moreover, the AUCs of R2*_whole_ for both observers and of R2*_largest_ for observer 1 were significantly higher than that of R2*_largest_ for observer 2 (p = 0.84/0.01 for R2*_largest_ vs R2*_whole_ [observer 1/observer 2], p<0.01 for R2*_whole_ [observer 1] vs R2*_largest_ [observer 2] and p = 0.86 for R2*_whole_ [observer2] vs R2*_largest_ [observer 1]). The performance of both R2*_largest_ and R2*_whole_ in differentiating ccRCC from renal masses other than ccRCC was ranked as ‘poor’ for AUCs smaller than 0.7. To discriminate high-grade from low-grade ccRCCs, both R2*_whole_ and R2*_largest_ were deemed as good (R2*_whole_: 0.70/0.72; R2*_largest_: 0.73/0.71[obserber1/observer2]) and were not significantly different (p = 0.89/0.93 for R2*_largest_ vs R2*_whole_ [observer 1/observer 2], 0.96 for R2*_whole_ [observer 1] vs R2*_largest_ [observer 2] and 0.96 for R2*_whole_ [observer 2] vs R2*_largest_ [observer 1]).

**Table 3:** The diagnostic performance of R2*_largest_ and R2*_whole_ in evaluation of renal masses.

	R2*_largest_				R2*_whole_			
	Cut-off value	AUC	Sensitivity	Specificity	Cut-off value	AUC	Sensitivity	Specificity
**Observe 1**								
**RCC vs Benign lesion**	34.28	0.75	75.02	63.26	39.94	0.79	71.83	77.82
**HG** ^a^ **vs LG** ^b^	35.08	0.73	60.39	79.32	33.60	0.70	62.74	77.11
**ccRCC vs other type** ^c^	34.74	0.59	60.14	53.28	36.98	0.57	40.00	86.96
**Observe 2**								
**RCC vs Benign lesion**	33.52	0.64	31.25	96.70	37.67	0.78	68.50	78.81
**HG vs LG**	29.26	0.71	57.26	80.95	36.85	0.72	51.61	85.11
**ccRCC vs other type**	29.15	0.53	41.43	73.91	34.97	0.59	42.86	85.06

^a^. high grade of ccRCC.

^b^. low grade of ccRCC.

^c^. renal masses other than ccRCC.

## Discussion

BOLD MRI reflects tissue information that is dependent on the level of blood oxygenation within the tissue components [13, 14]. A previous study reported on the difference in R2* values in different renal masses [15]. Based on the results of the former study, we used BOLD MRI as a non-invasive technique to differentiate RCC from benign renal masses. Both the R2*_largest_ vs R2*_whole_ were significantly higher for benign renal masses than for RCC. Generally speaking, the presence of deoxyhaemoglobin creates magnetic susceptibility perturbations around blood vessels, thereby increasing the transverse MR relaxation rate (R2*) of the surrounding tissue in proportion to the tissue deoxyhaemoglobin concentration [16]. In agreement with a previous study [14], the results of our study show that the R2* values in benign lesions (mainly comprising AMLs) were significantly higher than those of RCCs. AMLs comprised a variable mixture of fat, smooth muscle, and abnormal blood vessels [17]. We assumed that the components of AMLs such as mature adipose tissue may be associated with decreased blood volume, blood flow, or oxygen consumption, which may lead to an elevated deoxyhaemoglobin concentration and contribute to the increase in R2* values.

The diagnostic value of R2* in evaluating renal masses and the effect of the parameter approach based on different image sections were not clearly explored. Former studies used the R2* value as a research parameter and showed that R2* values of solid lesions were significantly higher than those of benign cystic lesions. However, the diagnostic value of the R2* value in differentiating RCC from benign renal masses ranked as “very good” when using R2*_whole_ and R2*_largest_ for observer 1, whereas R2*_largest_ for observer 2 did not. This result indicates that although the parameter may be significantly different in some groups according to the Mann-Whitney U test, it may not be good enough to be used as a diagnostic tool in clinical practice. Moreover, our study showed that the inter-observer agreement of parameter measurements based on the whole tumour volume was superior than that based on the largest cross-section, and the R2* value derived from the largest cross-section may not be sufficiently proficient in the evaluation of renal masses based on the different results with each observer. We believe that for analysis based on the whole tumour volume instead of the largest cross-section, the parameter measurement may be more representative of the tumour with an increased measurement area and frequency, and we believe that good repeatability and reproducibility in the calculation should be an important element in clinical application and could contribute to the diagnostic results in differentiating RCC from benign renal masses.

The importance of evaluating the ccRCC grade before treatment is well recognised. RA and active surveillance are currently accepted as optional treatment approaches for RCC [18,19]. Former studies have reported that the histopathological features of ccRCC are crucial in determining whether RA or active surveillance is optional during the treatment process [20]. The Fuhrman grade classification can help determine the histopathological features of ccRCCs and predict the prognosis of patients based on the microscopic morphology of a neoplasm. In a recent study, BOLD MRI was shown to be useful in differentiating a hypervascular mass from a hypovascular one [21], and it was also shown to be related to the Fuhrman grade. Here, we demonstrated the potential usefulness of BOLD MRI in grading ccRCCs and revealed that there were significant differences in the R2*_largest_ and R2*_whole_ values of low- and high-grade ccRCCs. The R2*_largest_ and R2*_whole_ values of dominant structures were significantly higher for high-grade ccRCCs than for the lower-grade ccRCCs. We believe that these data are in accordance with former results, which showed that more intratumoural vascular structures were present in low-grade tumours than in high-grade ccRCCs and led to the different oxygen level in the tumour tissue [22,23]. Therefore, we believe that BOLD MRI is useful in grading ccRCCs and could be a noninvasive tool for tumour grading.

The results of previous studies [24] have suggested that patients with chromophobic or papillary RCC have a better prognosis than patients with ccRCC. In addition, particularly in patients with advanced and metastatic RCCs, these subtypes respond differently to molecularly targeted therapies. The identification of RCC subtypes is an important goal for imaging evaluation. Some previous studies have proven to be useful in differentiating RCC subtypes using CT, MR DWI, and others [25,26], and a recent study showed that for characterising RCC subtypes, DWI and BOLD MRI at 3 T may be useful but that the current BOLD MRI technique seems to have a limited diagnostic accuracy [27]. In our study, we also assessed the ability of the R2* value to differentiate ccRCC from renal masses other than ccRCC rather than from subtypes of RCC because we believe that the former is closer to the practical situation in clinical procedures, and we believe that discriminating ccRCC from renal masses other than ccRCC is clinically important. Similarly to a previous study [27], BOLD MRI was not sufficiently proficient in distinguishing ccRCC from renal masses other than ccRCC. Although the cut-off values of R2*_largest_ and R2*_whole_ could be determined through ROC analysis, the diagnostic value was relatively poor according to the AUC. As renal masses other than ccRCC have varied histopathological types, leading to an extensive range of R2* values, the large overlap of R2* values between ccRCC and renal masses other than ccRCC was the main explanation for our results.

There are several limitations to this study. First, our study was limited to a single institution. Although our study shows the promising usefulness of R2* values derived from the whole tumour in evaluating renal masses, our results still need to be verified by larger studies. Second, the effect of haemodynamics on renal lesions was not clarified and will need to be studied further to improve our understanding and to interpret the results of our study. Third, the intra- and inter-observer variability was good when calculating the R2* values according to our study. However, ROIs were still manually placed, which may be affected by the diversity between observers with different levels of experience and knowledge. More dedicated software should be developed and used for measuring R2* values in the kidney and will be needed in future studies and applications. Moreover, some benign renal masses were not confirmed histopathologically, but they did undergo periods of imaging follow-up for at least 18 months. Without histological confirmation, there was no exact way to confirm the benign renal lesions. Therefore, no change in the size of the lesions during the relatively long-term follow-up of at least 18 months might be considered as an alternative method. In our study, we use different cut-off values in the ROC analysis to reach the highest diagnostic value for each reader. However, regarding the clinical procedure, with the results of intra-observer and inter-observer agreements, it might be more useful and reasonable for future research to propose a single cut-off. Finally, as contrast-enhanced CT or MRI is the gold standard in imaging examination, research to evaluate the usefulness of BOLD imaging combined with these conventional methods is needed to explore the additional value in the clinical setting.

In conclusion, the application of BOLD imaging could provide an alternative to invasive methods for differentiating renal cell carcinoma from benign renal masses and for predicting clear-cell renal cell carcinoma grading while providing a comprehensive pretreatment imaging evaluation. Assessing the whole tumour provides better inter-observer agreement in parameter measurement for differentiating renal cell carcinoma from benign renal masses compared with assessing the largest cross-section.
